# Radiologic and Pathologic Insights into Medication-Related Osteonecrosis of the Jaw in Myeloma Patients: A Report of 3 Cases

**DOI:** 10.1016/j.radcr.2025.06.109

**Published:** 2025-07-24

**Authors:** Suvarna Indermun, Jessica Simpson, Kim Pedro-Beech, Julandi Alwan, Leon Janse van Rensburg

**Affiliations:** a Department of Craniofacial Biology, Pathology and Radiology, Faculty of Dentistry and WHO Collaborating Centre, University of the Western Cape, Cape Town, South Africa; b Department of Maxillofacial and Oral Surgery, Faculty of Dentistry and WHO Collaborating Centre, University of the Western Cape, Cape Town, South Africa; c Department of Anatomical Pathology, National Health Laboratory Service, Tygerberg Hospital; d Department of Medical Imaging and Clinical Oncology, Faculty of Medicine and Health Sciences, Stellenbosch University, Cape Town, South Africa

**Keywords:** MRONJ, Bisphosphonates, Panoramic radiography, Maxillofacial pathology, Bone necrosis, Malignancy

## Abstract

Medication-related osteonecrosis of the jaw (MRONJ) is a rare side-effect of certain drugs, mainly those used in regimens treating specific cancers and in the initial treatment for non-neoplastic conditions. The first cases of jaw osteonecrosis were reported over 2 decades ago in patients taking bisphosphonates (BPs). Since its identification, the nomenclature has changed from bisphosphonate-related osteonecrosis of the jaw (BRONJ) to MRONJ, as other drugs can elicit the same reaction. Invasive dental treatments, such as extractions, are known risk factors. Therefore, the importance of routine dental care before and during such drug therapy cannot be overemphasized. MRONJ is a challenging condition to diagnose early as the radiographic features are subtle and easily overlooked. This case report presents 3 female patients with multiple myeloma, emphasizing the chronological radiologic evolution of MRONJ. Notably, 1 case illustrates a transition from Stage 0 to Stage 2 over a 17-month period, documented through serial panoramic radiographs and a positron emission tomography (PET) scan. Another case highlights the role of cone beam computed tomography (CBCT) in identifying sequestra and pathological fractures. An additional noteworthy case illustrates that conservative dental management avoided the potential development of MRONJ. While histopathology confirmed necrotic bone in 2 cases, early radiologic indicators such as sclerosis and nonhealing sockets provided key diagnostic insights. This case report underscores the importance of regular dental monitoring and interdisciplinary care in at-risk patients and contributes to the growing discourse on the critical role of radiologic evaluation in the early detection and management of MRONJ.

## Introduction

Osteonecrosis of the jaw (ONJ) is still a relatively newly discovered complication of anti-resorptive drug therapy. Literature concerning this disease process was virtually nonexistent before 2003 [1], and since 2007, has risen progressively to approximately 200 articles per year. The first cases of ONJ were reported by Robert Marx in 2003, who attributed it to bisphosphonate use [[2], [3], [4]]. In his initial report, Marx mentioned 36 cases of painful bone exposure in the jaws that failed to respond to treatment. These patients were all receiving BP therapy (zoledronate or pamidronate). This finding represented an unrecognized and serious side-effect that raised safety concerns related to the prolonged use of such drugs. This led to a proliferation of studies that called for caution when prescribing BP’s [[5], [6], [7]].

Osteonecrosis refers to “the death of bone resulting from a transient or permanent disruption of blood supply to the bone” [8]. It is essential to differentiate MRONJ from other causes of delayed healing, which led to the subsequent modification of the working definition of MRONJ in the 2022 American Association of Oral and Maxillofacial Surgeons (AAOMS) position paper [8]. Patients are considered to have MRONJ if *all* the following characteristics are present: (1) current or previous treatment with antiresorptive therapy alone or in combination with immune modulators or anti-angiogenic medications, (2) exposed bone or bone that can be probed through an intraoral or extraoral fistula(e) in the maxillofacial region that has persisted for more than 8 weeks, and (3) no history of radiation therapy to the jaws or metastatic disease to the jaws [1,8].

Bone-modifying agents, such as BP’s and denosumab, are prescribed for skeletal disorders, including osteoporosis, bone metastases, and multiple myeloma [1]. Patients at risk for, or diagnosed with, MRONJ may have other underlying health conditions that can mimic its presentation. Clinicians should take these potential differential diagnoses into account when evaluating a suspected case of MRONJ. Conditions often misdiagnosed may include but are not limited to periodontitis, sinusitis, alveolar osteitis, caries, periapical pathology, chronic osteomyelitis, atypical neuralgia, fibro-osseous lesions, sarcoma, and temporomandibular joint disorders [9].

Whilst a histological component is not yet part of the definition, histological examination of the resected bone segment is only advised to rule out metastases in the jaws, since a majority of MRONJ patients suffer from underlying malignancy [7]. There are insufficient distinctive histological features to differentiate between osteomyelitis, MRONJ and osteoradionecrosis (ORN), making a final diagnosis purely based on histology impractical [10].

Staging of MRONJ is carried out according to the AAOMS guidelines (Table 1) and is based on the severity of the symptoms and the scope of the clinical and radiographic findings [1,4,8,9,11].

**Table 1:** Stages of MRONJ [1,7,8].

Category	Description
At risk	No apparent necrotic bone in asymptomatic patients who have been treated with IV or oral antiresorptive therapy.
Stage 0	Patients with no clinical evidence of necrotic bone but who present with nonspecific symptoms or clinical and radiographic findings, such as:
	Symptoms:
	• Odontalgia not explained by an odontogenic cause. • Dull, aching bone pain in the jaw, which may radiate to the temporomandibular joint region. • Altered neurosensory function.
	Clinical findings:
	• Loosening of teeth not explained by chronic periodontal disease. • Intraoral or extraoral swelling.
	Radiographic findings:
	• Alveolar bone loss or resorption not attributable to chronic periodontal disease. • Changes to trabecular pattern sclerotic bone and no new bone in extraction sockets. • Regions of osteosclerosis involving the alveolar bone and/or the surrounding basilar bone • Thickening/obscuring of periodontal ligament (thickening of the lamina dura, sclerosis, and decreased size of the periodontal ligament space).
Stage 1	Exposed and necrotic bone or fistula that probes to the bone in patients who are asymptomatic and have no evidence of infection/inflammation. these patients also may present with radiographic findings mentioned for Stage 0 that are localized to the alveolar bone region.
Stage 2	Exposed and necrotic bone or fistula that probes to the bone, with evidence of infection/inflammation. these patients are symptomatic. these patients also may present with radiographic findings mentioned for Stage 0 localized to the alveolar bone region.
Stage 3	Exposed and necrotic bone or fistulae that probes to the bone, with evidence of infection, and 1 or more of the following:
	• Exposed necrotic bone extending beyond the region of alveolar bone (i.e. inferior border and ramus in the mandible, maxillary sinus and zygoma in the maxilla). • Pathologic fracture. • Extraoral fistula. • Oral antral/oral-nasal communication • Osteolysis extending to the inferior border of the mandible or sinus floor.

The pathogenesis of MRONJ is still not fully understood and remains unclear. Several theories have been postulated with regard to the aetiology of MRONJ, including reduced jaw remodeling rate, angiogenesis inhibition, soft tissue toxicity, and immune system dysfunction [11]. Several studies have indicated that dental extraction and/or infection was the initiating factor [1,8,9,12].

The risk for osteonecrosis is low, but its existence should dictate the judicious use of such agents. The incidence of MRONJ is reported as 1% in cancer patients and 0.1% in patients with metabolic bone disease [13]. Osteonecrosis can be debilitating with several negative side-effects, such as persistent jaw pain, chronic infections, difficulty eating and chewing, pathological fractures, psychosocial impact, and the potential need for invasive surgical interventions [14]. However, the therapeutic advantages of these drugs outweigh the risks. To date, stabilization rather than cure is the only means of treatment [9,11,15,16].

In this paper, 3 cases of multiple myeloma are presented, with 2 positive for MRONJ. Case 1 illustrates a preferred scenario where conservative management of the offending teeth prevented the progression to necrosis. An extraction in this instance *could* have resulted in MRONJ. This practical illustration underscores the importance of radiological assessment in MRONJ, reiterating the aim of the AAOMS position paper [8].

The aim of this report is to illustrate the spectrum and progression of MRONJ through radiographic and histopathologic correlation, to emphasize the importance of serial imaging, and to highlight how early intervention strategies can influence patient outcomes. In doing so, this paper adds to the growing call for integrating radiologic surveillance into MRONJ staging and management protocols.

(*Note teeth are numbered using the FDI classification.)

### Case 1

**Fig. 1:**
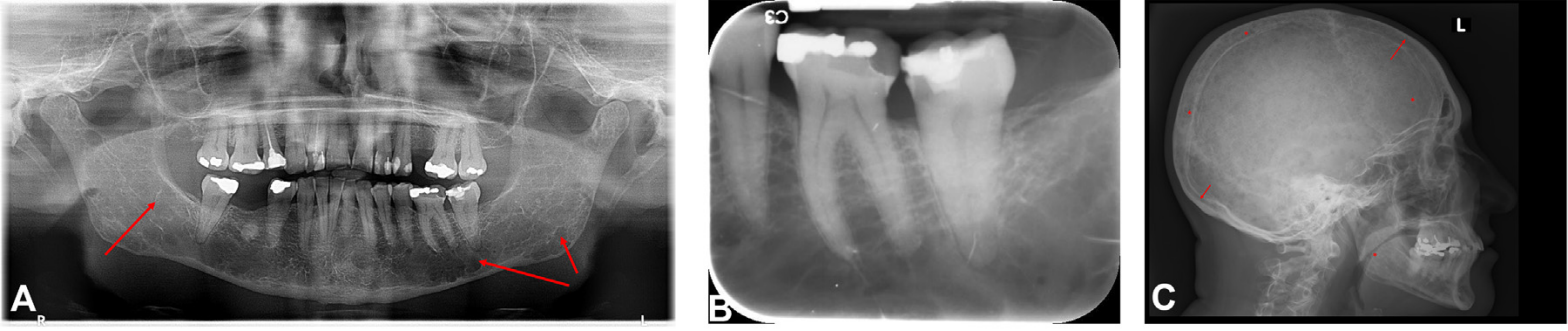
Case 1: (A) Panoramic radiograph showing multiple, well-defined lucencies located bilaterally, extending from the angles of the mandible to the ascending rami (red arrows). Both inferior alveolar nerve canals lack cortication. (B) Periapical radiograph of #36 and 37 showing compromised restorations. (C) Lateral skull view showing multiple small, lucent lesions involving the entire skull and mandible (red asterisk), demonstrating endosteal scalloping (red arrows) and lytic areas.

A 61-year-old female, known with diabetes mellitus, presented with a main complaint of pain on teeth #36 and #37. The panoramic radiograph (Fig. 1A) revealed extensive restorative work with compromised restorations on teeth #36 and #37. Tooth #37 showed mesio-occlusal caries, with an apical lucency and accompanying periodontal ligament widening (Fig. 1B).

An incidental finding of multiple well-defined lucencies were located bilaterally, extending from the angles of the mandible to the ascending rami. These lesions were more extensive on the left side. A lateral skull view (Fig. 1C) showed multiple small, lucent lesions involving the entire skull and mandible, demonstrating endosteal scalloping and lytic areas, consistent with multiple myeloma.

The patient’s medical history revealed that she was diagnosed with plasma cell myeloma 4 months prior and was undergoing treatment with cyclophosphamide, dexamethasone and thalidomide. Thalidomide was stopped shortly after due to side effects and was to be restarted in preparation for a possible stem cell transplant. Although symptomatic, extraction was deferred due to her recent myeloma diagnosis and ongoing systemic therapy. Conservative dental treatment was planned for teeth #36 and #37, namely a replacement of the restoration and endodontic treatment respectively, to mitigate the risk of MRONJ development. Considering her medical history and the radiological findings, this patient was classified as being at risk for MRONJ.

### Case 2

A 45-year-old female, with a medical history of refractory multiple myeloma of the pelvis and Type 2 diabetes mellitus, presented with pain related to the lower right molar region. Her current treatment consisted of dexamethasone (40 mg weekly) and bi-weekly chemotherapy sessions. She had a history of tooth extractions of the #22, and #48. Over 17 months, the patient returned with a complaint of continued pain and nonhealing of the 48-extraction socket. Notably, the 22-region showed progressive healing.

**Fig. 2:**
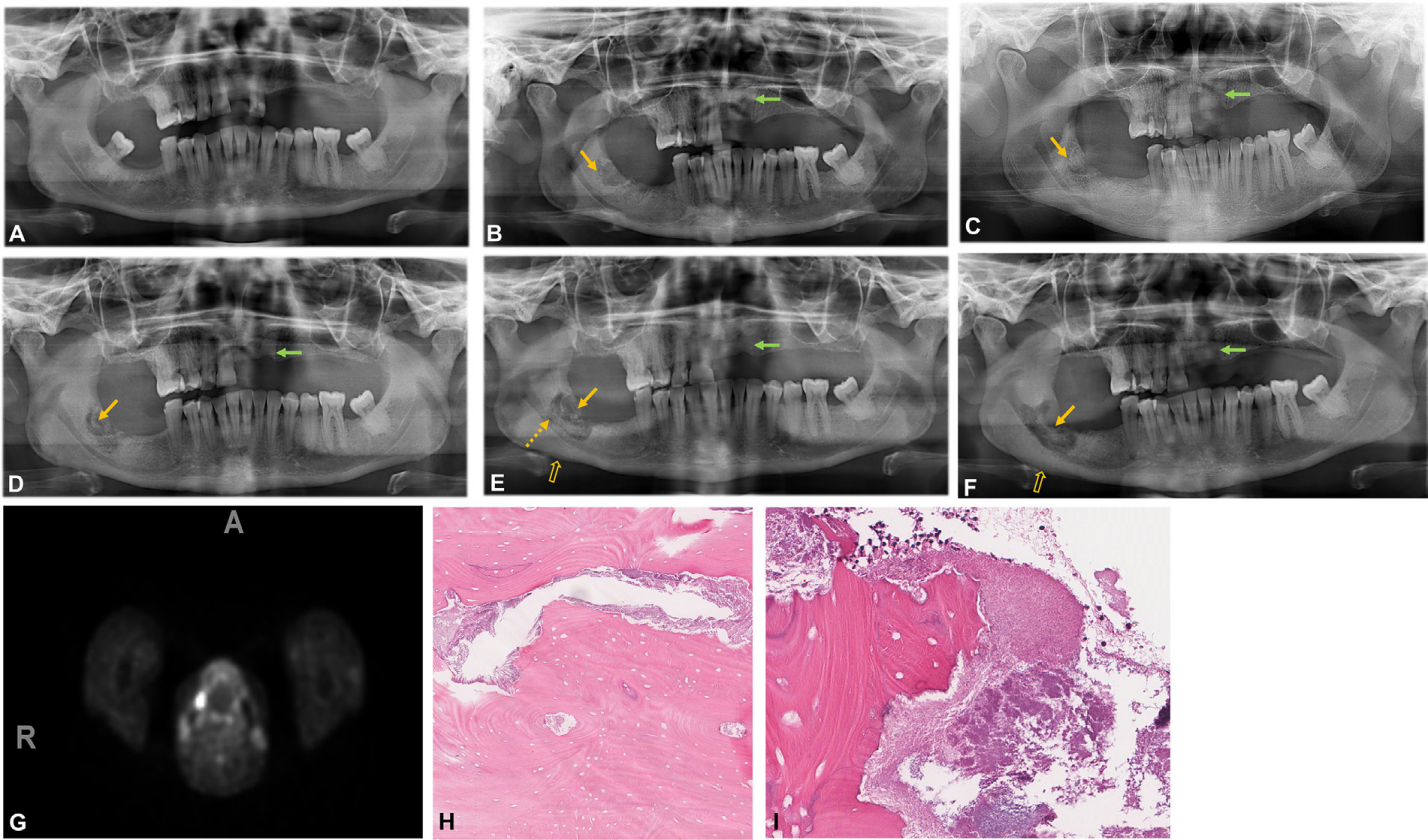
Case 2: (A) May 2019. First dental visit. A panoramic radiograph shows the jaws are partially edentulous. There is mild to moderate generalized horizontal periodontal bone loss. Caries was present on the #16, #15, #12, and #48. A defective restoration noted on the #22. (B) October 2019. Teeth #22 (green arrow) and #48 (orange arrow) were extracted 4 months previously. The extraction sockets are visible. The #48 socket shows an incomplete inferior sclerotic margin. (C) February 2020. Tooth #22 had undergone healing (green arrow). The #48 region (orange arrow) shows a mixed radiolucent and sclerotic appearance. The incomplete inferior sclerotic margin is surrounded by a faint lucent line, raising concern of sequestra formation. (D) March 2020.The #48 region shows progressive enlargement of mixed lytic and sclerotic lesion. The sclerotic bone fragments are consistent with sequestration. (E) May 2020. The #48 region shows progressive interval enlargement. There is an increase in the size and number of sclerotic bone fragments (closed orange arrow), consistent with sequestra, cortical destruction and erosion of the superior border of the inferior alveolar nerve canal (dashed orange arrow). A periosteal reaction was evident at the inferior mandibular cortex (open orange arrow). The #22 region had healed (green arrow). (F) September 2020: Progressive enlargement of the mixed lytic and sclerotic # 48 lesion with sequestrum formation. Progression of the periosteal reaction can be seen (open orange arrow), giving the appearance of “onion skin.” (G) October 2020 F-18 FDG PET/CT showing increased FDG uptake in the right mandibular molar region related to the known extraction. (H) Empty lacunae characterize nonvital bone, with irregular surface resorption (H&E, x75). (I) Abundant basophilic bacterial colonies and neutrophils occupying the irregular bone surface outline caused by resorption (H&E, x200).

Serial clinical and radiographic follow-up, including panoramic radiographs, revealed a nonhomogeneous lesion extending from the alveolar ridge to the superior aspect of the inferior alveolar nerve canal in the #48 region. The lesion measured 28.7×16.9 mm and displayed mixed lytic and sclerotic areas, raising differential diagnoses of osteomyelitis, osteonecrosis, and alveolar osteitis (Fig. 2A–F). Table 2 summarizes the imaging timeline.

**Table 2:** Summary of radiographic findings.

Case	Age/Sex	Systemic condition	Imaging modalities	Key radiographic findings	MRONJ stage	Histopathology
1	61/F	Multiple myeloma, diabetes	Panoramic radiograph, Periapical	Apical lucency, bilateral mandibular lucencies, skull lesions	At-risk (No MRONJ)	Not performed
2	45/F	Multiple myeloma diabetes	Serial Panoramic radiographs, PET/CT	Persistent socket, mixed lucent-sclerotic lesion, periosteal reaction, sequestra	Stage 0 → Stage 2	Confirmed necrotic bone
3	60/F	Multiple myeloma	Panoramic radiograph, CBCT	Moth-eaten appearance, sequestra, pathological mandibular fracture	Stage 3	Confirmed necrotic bone

A fludeoxyglucose-18 (FDG) positron emission tomography (PET) scan showed no new myeloma lesions. However, avid FDG increased uptake in the right mandibular molar region corresponded to the known extraction site (Fig. 2G). Follow-up bone marrow biopsies and debridement showed poor responses to therapy. The histopathological sections (Fig. 2H and I) were nonspecific showing nonvital bone with irregular surface resorption with abundant basophilic surface bacterial colonies and acute inflammation, with no evidence of malignancy. The patient subsequently demised due to medical complications.

This patient was initially categorized as Stage 0 MRONJ, as she presented with nonspecific clinical and radiographic findings but ultimately progressed to Stage 2 at the time of diagnosis.

### Case 3

A 60-year-old female presented with a pathological right femur fracture. As the patient had no known co-morbidities at the time and an osseous biopsy was performed, revealing plasmacytoma. Serum Protein Electrophoresis (SPEP), Urine Protein Electrophoresis (UPE), and immune fixation revealed a small M-peak. Kidney function and calcium levels were all within normal range. A skeletal survey showed multiple lytic lesions. A bone marrow aspiration and analysis revealed an increased number of plasma cells (81%) with marked atypia, as well as reduced tri-lineage hematopoiesis. The diagnosis of multiple myeloma was confirmed.

Initial treatment included cyclophosphamide and dexamethasone. Six months later, a bone marrow biopsy was requested to assess the response to treatment. The bone marrow aspirate revealed plasma cells less than 1% of all nucleated cells, consistent with a favorable treatment response.

**Fig. 3:**
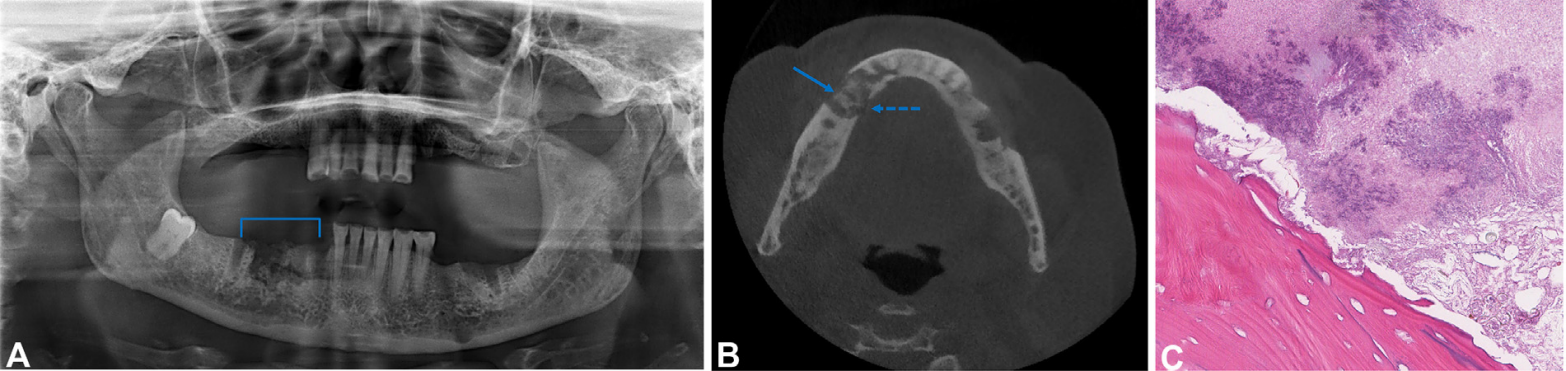
Case 3: (A) The panoramic radiograph revealed a moth-eaten appearance associated with the extraction sockets of the missing teeth in the second, third, and fourth quadrants, particularly the #43-46 teeth (blue bracket). (B) Axial CBCT image showing multiple, irregular, mixed lytic and sclerotic bone fragments consistent with sequestra formation (blue arrow) at the #43 and #44 tooth sockets and a pathological fracture (blue dashed arrow). (C) Nonvital bone with an irregular, scalloping surface outline, colonized by basophilic bacteria (H&E, x100).

However, after 2 years of follow-up, the patient presented with a main complaint of nonhealing sockets following multiple tooth extractions (Fig. 3A and B). The clinical history and radiographic features were consistent with the diagnosis of MRONJ as the patient had no clinical evidence of necrotic bone but presented with aching bone pain in the jaw. The axial CBCT confirmed the presence of sequestra formation and a pathological fracture, categorizing the patient as stage 3.

Histopathologic assessment (Fig. 3C) of both the mandibular and maxillary bone biopsies revealed the presence of nonvital bone with an irregular surface outline due to resorption. Abundant basophilic bacteria colonized the bone surface, with evidence of acute inflammation. No features concerning plasma cell neoplasm were observed. The patient subsequently died due to medical complications.

This case was classified as Stage 3 MRONJ, characterized by extensive radiologic destruction, confirmed sequestrum, and a pathological fracture. The use of CBCT provided high-resolution detail, which allowed accurate assessment of the lesion extent and confirmed diagnosis.

## Discussion

Table 3 summarizes the radiologic and pathological spectrum of all 3 cases. This radiologic review of 3 female myeloma patients underscores the critical need for heightened awareness and early detection of MRONJ. A systematic review by Frutuoso et al. found that females are more frequently affected by MRONJ than males, with a ratio of 2.49:1 [4]. This aligns with existing literature, which attributes the higher prevalence in females to underlying conditions such as osteoporosis and breast cancer, both of which are more common in women. The average age at diagnosis was 66.27 years, with the 60 to 70-year age group being the most affected. This finding also supports the literature, which identifies age over 65 years as a significant risk factor for MRONJ [4,8,17].

**Table 3:** Chronological imaging timeline of case 2.

Date	Clinical event	Imaging	Main findings	MRONJ stage
May, 2019	Initial dental visit	Panoramic radiograph	Caries #22, #48, mild periodontal loss	At risk
October, 2019	Postextraction radiograph	Panoramic radiograph	#22 healed, #48 socket unhealed	Stage 0
February, 2020	Persistent symptoms	Panoramic radiograph	Mixed lesion at #48, early sequestrum suspected, Healing #22 region	Stage 0
March, 2020	Worsening symptoms	Panoramic radiograph	Enlargement, more sequestra	Stage 0
May, 2020	Continued deterioration	Panoramic radiograph	Cortical erosion, periosteal reaction, enhancement of right inferior alveolar canal	Stage 1 progressing
September, 2020	Further progression	Panoramic radiograph	Onion-skin periosteal reaction, lesion expansion	Stage 2 suspected

The AAOMS reported on evidence that patients with multiple myeloma receiving anti-angiogenic and anti-resorptive drugs have a higher MRONJ prevalence [8]. Co-morbid conditions and medication associated with immunosuppression are considered independent risk factors for MRONJ [8]. All 3 cases involved female patients with multiple myeloma, with treatment regimens including the corticosteroid, dexamethasone. All patients had compromised oral hygiene. The patients in Case 2 and Case 3 had no clinical evidence of necrotic bone but presented with aching bone pain in the jaw, and notably no new bone in the extraction sockets. Regions of osteosclerosis involving the alveolar bone and obscuration of the periodontal ligament space were also noted.

### Radiological detection and progression

The radiographic changes seen in MRONJ include persisting extraction sockets, thickening of the lamina dura, widening of the periodontal ligament space, thickening of the mandibular cortex, regional osteosclerosis, enhancement of the inferior alveolar canal and sequestrum formation [1,17,18]. Several studies have reported that conventional 2D images are of limited diagnostic value, as they can underestimate the extent of changes in the bone. Cone beam computed tomography (CBCT) has a greater value for detecting early asymptomatic lesions in the jaws [17].

Case 2 uniquely demonstrates radiographic progression from Stage 0 to Stage 2 MRONJ over a 17-month period. The sequential panoramic radiographs clearly document evolving radiologic changes including the development of mixed sclerotic and lytic bone, sequestrum formation, and periosteal reactions. This timeline of progression supports findings that emphasize the diagnostic value of persistent sockets, cortical irregularities, and sclerotic changes as early indicators of MRONJ [17,19,20].

Case 2 was initially classified as Stage 0. Stage 0 is considered a prodromal and nonexposed state [1,8]. According to Moreno-Rabié et al., [17] this stage is still controversial because of its ambiguity and nonadherence with the definition. The controversy stems from the lack of exposed necrotic bone, which is a hallmark of MRONJ. Additionally, Stage 0 relies on nonspecific clinical and radiographic findings, such as bone pain, subtle osteosclerotic changes, and vague radiographic anomalies, which overlap with other maxillofacial conditions. These challenges make it difficult to differentiate Stage 0 MRONJ from other conditions like osteomyelitis or chronic alveolar osteitis, potentially leading to misdiagnosis and delayed intervention. For this reason, it is suggested that imaging would be instrumental in making a preclinical diagnosis. Devlin et al. also agrees and advises that waiting for bone exposure as a criterion for diagnosis of MRONJ will lead to delayed diagnosis and worse prognosis [21]. Moreno-Rabié *et al.'s* systematic review also reported a paucity of literature on the early radiographic findings [17]. Histopathologic analysis confirmed necrotic bone in Cases 2 and 3. These features, while supportive, lack diagnostic specificity and must be correlated with imaging and clinical findings.

### Mechanism of development and risk factors

In Case 2, the chronology of the patient's symptoms and radiographic findings raised critical questions about the different healing outcomes between the 2 extraction sites and the underlying factors influencing these differences. This case emphasizes the importance of considering the patient’s medical history in the diagnostic process, as well as anatomical factors influencing bone healing postdental extractions. It is thought that the jaw is more predisposed to MRONJ compared to other bones. This may be due to the increased vulnerability to bacterial infections, and that BPs are preferentially deposited in bone with higher turnover rates, such as the jaw [22]. MRONJ is also more likely to occur in the mandible than the maxilla, possibly due to the reduced blood supply of the mandible [4]. The mandible’s vascular supply, primarily dependent on the inferior alveolar artery, is critical to understanding its susceptibility to osteonecrosis. Reduced vascularity and localized infections amplify the risk, especially in this region of high turnover [8,19].

It has been shown that the majority of MRONJ cases are attributed to the administration of BP or denosumab for malignant diseases such as multiple myeloma, breast cancer and prostate cancer [7,8,20]. However, a significant proportion of cases occur due to chronic medication taken by patients with metabolic abnormalities, such as Paget disease and osteoporosis [7,20]. Pathologic osteoclast-mediated bone resorption is a characteristic trait of diseases such as metastatic breast and prostate cancer, multiple myeloma, and giant cell tumors of the bone. It can therefore be understood why osteoclast inhibition is a therapeutic goal in the treatment of such diseases. Drugs involved in MRONJ act on osteoclasts either directly or indirectly. BPs are a class of drugs that act exclusively on bone, to prevent loss of bone density. They are called ‘bisphosphonates’ because they have 2 phosphonate groups. The mechanism of action involves the inhibition of bone resorption by targeting osteoclasts and their precursor cells. The reduction in bone resorption is accompanied by a positive calcium balance [20], resulting in the radiographic appearance of a more radiopaque jaw [21,23].

Inhibition of bone remodeling, angiogenesis, and immune dysfunction, contribute to the development of MRONJ. The initiation and progression of multiple myeloma is driven by various mutations in different pathways that disrupt the intrinsic biology of plasma cells, altering them in ways that give rise to the characteristic features of myeloma [24]. While patients can have a genetic predisposition for diseases such as multiple myeloma, it is important to clarify that MRONJ is not a genetic condition. However, a recent study by Bojtor et al., has found that single nucleotide polymorphisms (SNPs) in genes like SIRT1, VEGFA, and CYP2C8 can play a role in MRONJ susceptibility. Established risk factors, together with newly identified genetic predispositions, could be utilized to create a personalized algorithm incorporating genetic diagnostics to identify patients at high risk for MRONJ [14]**.**

McGowan and Ivanovski conducted a study identifying the patient populations at risk of MRONJ and determining which medical and dental co-morbidities are significant risk factors for the development of MRONJ [23]. Their systematic review revealed that chemotherapy, corticosteroids and smoking were the most frequently reported medical risk factors. The AAOMS also reports that corticosteroids are associated with an increased risk for MRONJ, especially when given in conjunction with anti-resorptive drugs [8].

Dental risk factors include poor periodontal health and mandibular tooth extraction [25]. Several studies report that among patients with MRONJ, tooth extraction is cited as a predisposing event ranging from 62% to 82%. The typical presentation has been described as a “nonhealing” extraction socket or exposed jawbone that eventually progresses to the associated sequestrum formation [15,21].

### Management

It is recommended that potential candidates for anti-resorptive therapy undergo thorough dental examinations and routine radiographic examinations [[8], [17], [21], [25]]. Cases 2 and 3 resulted in debilitating outcomes for the patient, whereas Case 1 was a preferred scenario illustrating the importance of early interdisciplinary dental and radiologic assessment. An extraction in this instance could have resulted in MRONJ. The conservative dental management in a patient at high risk for MRONJ successfully averted surgical intervention. This supports the emphasis on preventative strategies found in recent case-based literature which advocates pretreatment dental evaluations and conservative protocols to reduce MRONJ incidence [20,26].

The treatment of MRONJ, whether medically and/or surgically, is difficult and without consensus, and thus emphasis should be placed on preventative strategies [11]. As such, patients on bisphosphonate or anti-resorptive therapies should receive regular medical and dental treatment. Due to the complexity of diagnosing and managing MRONJ, a coordinated approach from an interdisciplinary team consisting of dentists, oral and maxillofacial surgeons, radio-oncologists, pathologists, radiologists, and nuclear medicine physicians, is advised [27].

Treatment ranges from local wound care with Chlorhexidine mouth rinses, laser therapy and laser surgery, conservative surgery such as curettage, to extensive surgery such as decortication and resection of all necrotic bone with or without fluorescent light, plasma-rich concentrates, teriparatide prescription and pentoxifylline and tocopherol (PENTO protocol) with varying outcomes [15,27,28]. Surgical intervention should be done judiciously as it is challenging to achieve a healthy bone margin in compromised bone and the associated surgical trauma has the potential to cause further osteonecrosis [28].

Pentoxiphylline (a xanthine derivative used to improve blood flow to tissues) and Tocopherol (an antioxidant), used in combination is a well-known treatment modality for ORN. This has been implemented in MRONJ cases as a nonsurgical protocol or with minimal surgical intervention with some success [28].

Since dental extraction is the most common risk factor for MRONJ, a PENTOX protocol should be considered pre- and postoperatively to reduce the risk. In limited-resource surgical settings, such as the authors’ institution, these drugs are unavailable in state pharmacies and often unaffordable for the patients to purchase in private practice.

Overall patient health, such as diabetes management and oral health education are essential. Although no individual management strategy or collection of strategies will eliminate all MRONJ risks, it is recommended that patients should be referred to a multidisciplinary team consisting of experienced dentists for consultation as soon as the oncologists prescribe anti-resorptive therapy. Performing dental examinations and treatment before initiating anti-resorptive therapy is the most effective prevention method to decrease the incidence of MRONJ [15,26,29,30]. At the minimum, the dental examination should consist of clinical and panoramic radiographic examinations with individual periapical films where indicated. The aim of dental treatment is to prevent infections and thus avoid the need for potentially invasive dental procedures [29]. Regular communication between professionals is critical in ensuring proper patient management [15,29]. Proper oral hygiene practice and maintenance is also a simple but effective strategy that cannot be over-emphasized [15,30]. A continuous effort to educate patients, dentists, and medical professionals about the real risks associated with these anti-resorptive therapies and clinical prevention strategies can prevent the development of MRONJ [17,30].

## Conclusion

The pharmaceutical industry and medical community will continue the search for and the development of medications that can effectively treat metastatic cancers and simultaneously avoid complications like MRONJ. However, dental practitioners should be aware of the clinical and radiological signs of this condition. The importance of a dental review before starting patients on such treatments should not be underestimated, as patients most commonly develop MRONJ postdental extraction as seen in case 2 and 3.

This case report highlights the diagnostic value of radiologic surveillance in the early identification and staging of MRONJ. Serial panoramic radiographs, CBCT, and PET/CT were important in tracking lesion progression, identifying sequestra, and guiding interventions. Notably, conservative dental management in 1 case effectively prevented MRONJ onset, underscoring the importance of individualized treatment planning and multidisciplinary collaboration.

Taken together, these cases contribute unique visual documentation of MRONJ progression, emphasizes the preventative value of imaging, and supports the incorporation of routine dental and radiologic assessments into the standard care protocols for patients receiving antiresorptive or antiangiogenic therapies. This proactive intervention will help in the management and treatment of MRONJ effectively, with resultant improved patient outcomes and quality of life.

## Patient consent

Written informed consent was obtained from the patients and/or the next of kin for the publication of this case report.
